# Carbon Cloth Supported Nano-Mg(OH)_2_ for the Enrichment and Recovery of Rare Earth Element Eu(III) From Aqueous Solution

**DOI:** 10.3389/fchem.2018.00118

**Published:** 2018-04-18

**Authors:** Yinong Li, Chen Tian, Weizhen Liu, Si Xu, Yunyun Xu, Rongxin Cui, Zhang Lin

**Affiliations:** The Key Laboratory of Pollution Control and Ecosystem Restoration in Industry Clusters (Ministry of Education), School of Environment and Energy, South China University of Technology, Guangzhou, China

**Keywords:** Nano-Mg(OH)_2_, carbon cloth, composite, rare earth, recovery

## Abstract

Nano-Mg(OH)_2_ is attracting great attention as adsorbent for pre-concentration and recovery of rare earth elements (REEs) from low-concentration solution, due to its superior removal efficiency for REEs and environmental friendliness. However, the nanoparticles also cause some severe problems during application, including aggregation, blockage in fixed-bed column, as well as the difficulties in separation and reuse. Herein, in order to avoid the mentioned problems, a carbon cloth (CC) supported nano-Mg(OH)_2_ (nano-Mg(OH)_2_@CC) was synthesized by electrodeposition. The X-ray diffraction and scanning electron microscopy analysis demonstrated that the interlaced nano-sheet of Mg(OH)_2_ grew firmly and uniformly on the surface of carbon cloth fibers. Batch adsorption experiments of Eu(III) indicated that the nano-Mg(OH)_2_@CC composite maintained the excellent adsorption performance of nano-Mg(OH)_2_ toward Eu(III). After adsorption, the Eu containing composite was calcined under nitrogen atmosphere. The content of Eu_2_O_3_ in the calcined material was as high as 99.66%. Fixed-bed column experiments indicated that no blockage for Mg(OH)_2_@CC composite was observed during the treatment, while the complete blockage of occurred to nano-Mg(OH)_2_ at an effluent volume of 240 mL. Moreover, the removal efficiency of Mg(OH)_2_@CC was still higher than 90% until 4,200 mL of effluent volume. This work provides a promising method for feasible application of nanoadsorbents in fixed-bed process to recycle low-concentration REEs from wastewater.

## Introduction

Recent years, Rare earth elements (REEs) are considered to be irreplaceable critical dopants for advanced materials in high-tech applications (Alonso et al., 2012), such as luminescent (Zhang et al., 2018), catalysts (Li et al., 2017; Lin et al., 2017), permanent magnets (Mudryk et al., 2017), and sensor material (Willa et al., 2017), due to their special metallurgical, optical, and electronic properties (Dutta et al., 2016). With the development of the society and industry, the demand for the REEs is increasing all over the world (Dutta et al., 2016; Tansel, 2017). However, the REEs resource is in a serious shortage due to their low reserves and outputs from the natural minerals. Therefore, in order to relieve the environmental burden and ease the potential supply crisis, many secondary resources for recycling REEs are developed, including discarded REEs-containing solid waste (Maroufi et al., 2017; Tansel, 2017) and industrial wastewater (Binnemans et al., 2013; Wilfong et al., 2017), etc. Nevertheless, the REE concentrations in most of the secondary resources are as low as hundreds of ppm (Binnemans et al., 2013, 2015), which makes their enrichment and separation extremely difficult.

In view of the above issues, adsorption is regarded as an effective technology for the pre-concentration and recovery of REEs, owing to its simplicity, easy handling, sludge-free operation, and cost effectiveness (Liu et al., 2017; Qi et al., 2017; Wilfong et al., 2017). Compared with other adsorbents, nano-Mg(OH)_2_, an environmental-friendly material, exhibits rapid kinetics, and high efficiency (with a maximum adsorption capacity of 1,827 mg/g) toward a typical REE of Tb(III) at extremely low concentrations (Li et al., 2013). However, it should be pointed out that, like other nanoadsorbents, the small size of nano-Mg(OH)_2_ also caused some issues and difficulties in separation and reuse, including mass transfer and excessive pressure drops when applied in fixed bed or any other flow-through systems (Zhao et al., 2011). Moreover, the release of nanoparticles into the environment is also a possible risk to ecosystems and human health. These drawbacks strongly hinder the application of nano-Mg(OH)_2_ in the wastewater treatment (Zhao et al., 2011; Tesh and Scott, 2014).

An effective approach for overcoming the above bottlenecks is to fabricate nano-Mg(OH)_2_ onto supporting materials of larger size (Zhao et al., 2011; Tesh and Scott, 2014; Chen et al., 2016). The resultant nanocomposite is expected to retain the inherent properties of nano-Mg(OH)_2_, while the supporting material would provide higher mechanical strength and improve the dispersity of nano-Mg(OH)_2_ (Chen et al., 2016). In the previous study, common supporting materials include natural polymers (e.g., Chitosan, alginate; Xiao et al., 2015; Zhang L. et al., 2015; Kwon et al., 2016), inorganic materials (He et al., 2011; Chen et al., 2017), synthetic macromolecule materials (Chen et al., 2016; Yu et al., 2016; Zhang et al., 2017), and some carbon materials (e.g., graphene, carbon nanotubes, carbon cloth; Dimpe et al., 2017; Kumar et al., 2017; Tian et al., 2017; Xu et al., 2017). Despite plenty of supporting materials can be alternates for nanomaterials, very few of them were used to support nano-Mg(OH)_2_. Jia et al. (2014) reported that the nanofibrous membrane of PA6@Mg(OH)_2_, which was fabricated by electrospinning technique combined with hydrothermal strategy, exhibited preferable removal ability to Cr(VI). Xie et al. (2014) developed a novel modified diatomite adsorbent modified by dispersed magnesium oxide nanoflake for the remediation of eutrophic lakes by removing excess ${\text{PO}}_{4}^{4-}$. Li et al. (2011) synthesized a Mg(OH)_2_@reduced graphene oxide composite using *in-situ* chemical deposition method. The composite exhibited excellent adsorption effect to methylene blue. In the above studies, nano-Mg(OH)_2_ was loaded onto the surface of carriers by coprecipitation or hydrothermal method, which still caused some drawbacks such as complicated operation, poor controllability, and low synthesis efficiency of nano-Mg(OH)_2_.

Compared with the above methods, electrodeposition is a rapid, simple, and low-cost method without high-temperature treatment to fabricate the nano-composites (Lv et al., 2011; Liu T. et al., 2015). Currently, carbon cloth (CC) has been considered to be an ideal candidate for supporting materials by electrodeposition, owing to the following three advantages: (1) CC has abundant functional group, which can fasten the nanoparticles on its surface; (2) CC has excellent mechanical property, admirable resistance to the acid/alkali and environmental friendliness; (3) CC has superior electrical conductivity, which can control the morphology and the loading amount of nanoparticles during the synthesis. An et al. (2016) fabricated the honeycomb-shaped porous NiCo_2_O_4_ on electro-etched CC with strong adhesion by electrodeposition. The composites as a bind-free electrode display fast kinetics and superior electrochemical behavior controlled by the surface reaction. Fan et al. (2016) reported a highly flexible electrode synthesized by a facile *in-situ* electrodeposition of MnO_2_ and polypyrrole on CC. The composite showed superior electrochemical performance.

In this work, a nanocomposite was synthesized by electrodepositing Mg(OH)_2_ nanoparticles onto carbon cloth. Adsorption performance of the nano-Mg(OH)_2_@CC composite to Eu(III), a typical species of REE, was investigated via batch adsorption experiments. The properties of nano-Mg(OH)_2_@CC nanocomposites before and after adsorption were characterized by X-ray diffraction (XRD), scanning electron microscope (SEM), energy dispersive spectroscopy (EDS), and inductively coupled plasma(ICP). Furthermore, fixed-bed adsorption experiments were conducted to assess the enrichment of Eu(III) and the recovery of nano-Mg(OH)_2_@CC for the treatment of effluent containing the Eu(III).

## Experimental section

### Reagents and materials

All solutions were prepared using deionized water. Europium chloride hexahydrate (EuCl_3_·6H_2_O) was obtained from Aladdin (China). Magnesium sulfate (MgSO_4_·7H_2_O), magnesium nitrate [Mg(NO_3_)_2_·6H_2_O] and sodium hydroxide (NaOH) were obtained from Sinopharm Chemical Reagent Co. Ltd. (Shanghai, China). All of the chemicals were analytical grade and used without further purification. Carbon cloth was obtained from PHYCHEMi Co. Ltd. (Taiwan, China).

### Preparation of adsorbents

**Scheme 1:**
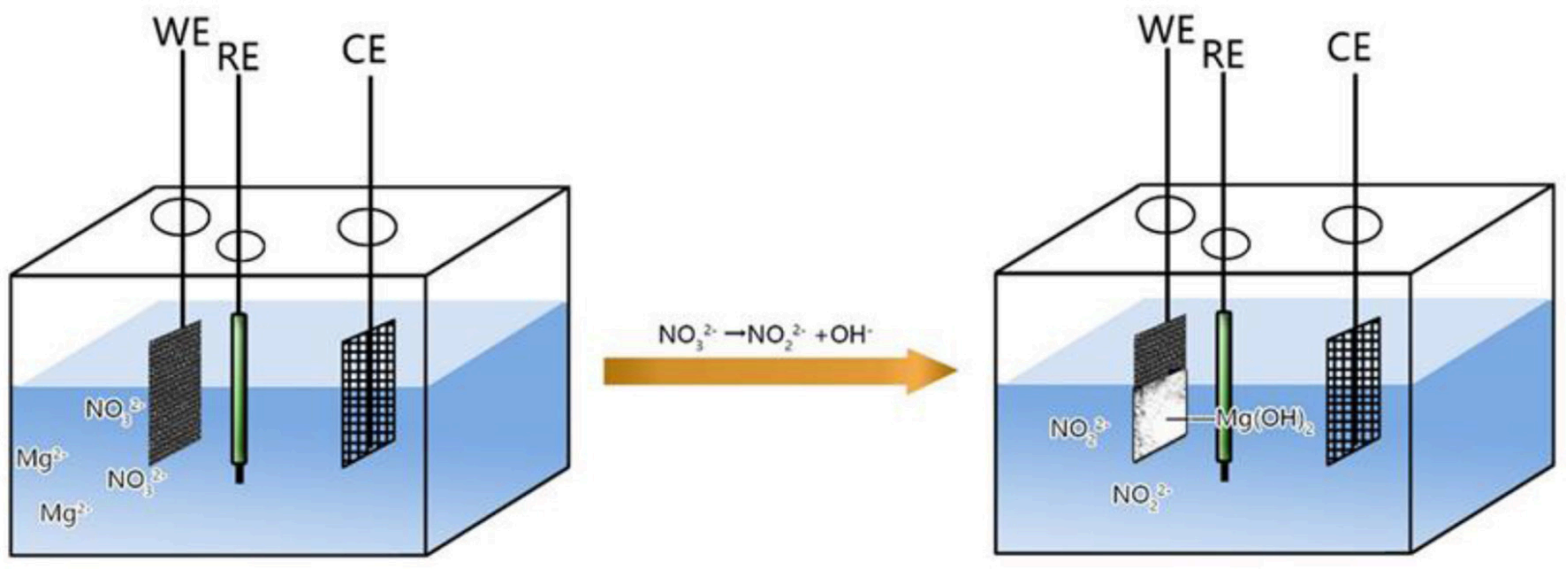
Schematic diagram showing the process for the engineering growth of the interlaced nano-sheet Mg(OH)2 on the surface of carbon cloth.

Flowerlike nano-Mg(OH)_2_ was prepared at room temperature (25°C) by precipitation from NaOH and MgSO_4_·7H_2_O solutions according to the method described in our previous study (Liu M. et al., 2015). The fabrication of Mg(OH)_2_@CC nanocomposite is illustrated in Scheme 1. In a typical process, a piece of commercial carbon cloth was washed by acetone, methanol, and isopropanol under sonication for 30 min to remove any contaminants on the surface. After drying in the oven, the piece of carbon cloth was cut into small rectangle piece of 2 ^*^1 cm and used as the working electrodes. However, only half of the CC was soaked into the electrolyte to make Mg(OH)_2_ only grow in an area of 1^*^1 cm. The electrodeposition of Mg(OH)_2_ on CC was conducted in a three-electrode system with a static potential of −1.5 V, soaking in Mg(NO_3_)_2_·6H_2_O aqueous solution of 1 mol/L. The counter and reference electrode utilized in the electrodeposition is platinum (Pt) net electrode and SCE electrode, respectively. After electrodeposition, the CC was rinsed by DI water and dried in a vacuum oven. Generally, the loading content of Mg(OH)_2_ on CC can be controlled by the electrodeposition time. Therefore, different electrodeposition times were conducted to optimize the preparation condition. Finally, the Mg(OH)_2_@CC nanocomposite prepared in optimal condition was used in the following study.

### Batch adsorption experiments

A stock solution of Eu(III) at a concentration of 1,000 mg/L was prepared using sodium Eu(III) (EuCl_3_·6H_2_O; AR). A series of 500 mL glasses of the solution were added to 200 mL of 10, 40, 70, 100, 130, 160 mg/L Eu(III) solutions and 14 mg Mg(OH)_2_/CC or 14 mg flowerlike nano-Mg(OH)_2_. The adsorbent quality used for analyzing the adsorption data of the two adsorbents are both in terms of the quality of magnesium hydroxide. The samples were shaken at 200 rpm at room temperature for 24 h to ensure the adsorption reaching equilibrium. The adsorption data were fitted using Langmuir (Equation 1) and Freundlich model (Equation 2). The Langmuir model expressed by the following equation:

(1) $$\begin{array}{r} \frac{C_{e}}{q_{e}}=\frac{C_{e}}{q_{m}}+\frac{1}{k_{L}q_{m}} \end{array}$$

Where *C*_*e*_ (mg/L) and *q*_*e*_ (mg/g) are the solute concentration and adsorption capacity at equilibrium, respectively, and *q*_*m*_ (mg/g) and *k*_*L*_ (L/mg) are the maximum monolayer adsorption capacity and the binding energy of adsorption, respectively. The Freundlich adsorption model expressed by the following equation:

(2) $$\begin{array}{r} q_{e}=k_{f}\;C_{\text{e}}^{1/\text{n}} \end{array}$$

Where *k*_*f*_ and n are the Freundlich constants measuring the adsorption capacity and the adsorption intensity, respectively.

Kinetic experiments were conducted by mixing certain amount of Mg(OH)_2_@CC or flowerlike nano-Mg(OH)_2_ into a 2,000 mL flask containing 100 mg/L Eu(III) solutions. For milli liter solution was sampled at various time intervals to determine the adsorption kinetics. The kinetic data were fitted by the pseudo-first-order (Equation 3) and pseudo-second-order equation (Equation 4):

(3) $$\begin{array}{r} q_{t}\;=\;q_{e}\;\left(1-e^{-k_{l}t}\right) \end{array}$$

(4) $$\begin{array}{r} q_{t}=\;\frac{q_{e}^{2}\;K_{2\;}\text{t}}{1+\;q_{e}{\;K}_{2\;}\text{t}} \end{array}$$

Where *q*_*e*_ (mg/g) is the adsorption capacity at equilibrium, *q*_*t*_ (mg/g) is the adsorption capacity at time *t*, and *k*_1_ (min^−1^) and *k*_2_ (g/mg·min) are the rate constants of pseudo-first-order and pseudo-second-order kinetics, respectively. The rate constants *k*_1_ and *k*_2_ were determined by plotting log(*q*_*e*_*-q*_*t*_) vs. *t* and *t/q*_*t*_ vs. *t*, respectively.

### Continuous effluent system experiments

Three grams nano-Mg(OH)_2_ was put into the sealed polytetrafluoroethylene groove with two cores to prevent the loss of nano-Mg(OH)_2_ (the inner groove size is 200 mm in length, 12 mm in width, 18 mm in height). The initial concentration of Eu(III) is 100 mg/L. The peristaltic pump was used to control the water inlet velocity, while the automatic collector was used for collecting samples at the same interval time. The flow rate is calculated by the volume of per unit time collected by the automatic collector. Figure S1a illustrates the above process in a simplified sequence flow diagram.

The composite loaded 3 g Mg(OH)_2_ was put into the sealed polytetrafluoroethylene groove with two slots to fix the Mg(OH)_2_@CC well into the groove (the inner groove size is 200 mm in length, 12 mm in width, 18 mm in height). The initial concentration of Eu(III) is 100 mg/L. The peristaltic pump was used to cycle 200 mL solution containing Eu(III) through the groove for 10 h with constant flow rate. The solution was replaced by 200 mL of fresh Eu(III) solution every 10 h, while each of the 200 mL solution was considered as one cycle to test the treatment effect of the nanocomposite to Eu(III). During processing for 25 circulations, the residual solution of every cycle was collected to measure the Eu(III) concentration. Figure S1b illustrates the above process in a simplified sequence flow diagram.

### Characterization

XRD patterns were collected on a Bruker X-ray powder diffractometer (advance D8) with Cu–Kα radiation. The tube voltage was 40 kV and the tube current was 40 mA. Diffraction patterns were collected over 2θ = 10°−80° at 1°/min. The step size of the scan was 0.02°. The morphology of the samples was observed using a JSM-7100F SEM with an Oxford INCA EDS. Eu(III) content in solution was determined by inductively coupled plasma optical emission spectrometry (ICP-OES).

## Results and discussion

### Mechanism study

Mechanism of electrodeposition process is shown in Scheme 1 and Equations (5–7):

(5) $$\begin{array}{rcl} NO_{3}^{-}+H_{2}0+2e^{-} & = & NO_{2}^{-}+2OH^{-}\;E^{\theta }=0.01V \end{array}$$

(6) $$\begin{array}{rcl} 2H_{2}O+2e^{-} & = & 2OH^{-}+H_{2}\;E^{\theta }=-0.83V \end{array}$$

(7) $$\begin{array}{rcl} Mg^{2+}+2OH^{-} & = & Mg{\left(OH\right)}_{2} \end{array}$$

Equations (5, 6) are the main electrode reaction equations of synthesizing nano-Mg(OH)_2_ by electrodeposition. Briefly, when the electrochemical workstation is in running state, the cathode will continuously produce the OH^−^ which combines with Mg^2+^ in solution, by electrolyzing ${\text{NO}}_{3}^{-}$ and H_2_O. When the concentration product of OH^−^ and Mg^2+^ reach the solubility product of Mg(OH)_2_, Equation (7) occurs and the Mg(OH)_2_ will adhere onto the surface of carbon cloth firmly.

### Characterization of the adsorbents

**Figure 1:**
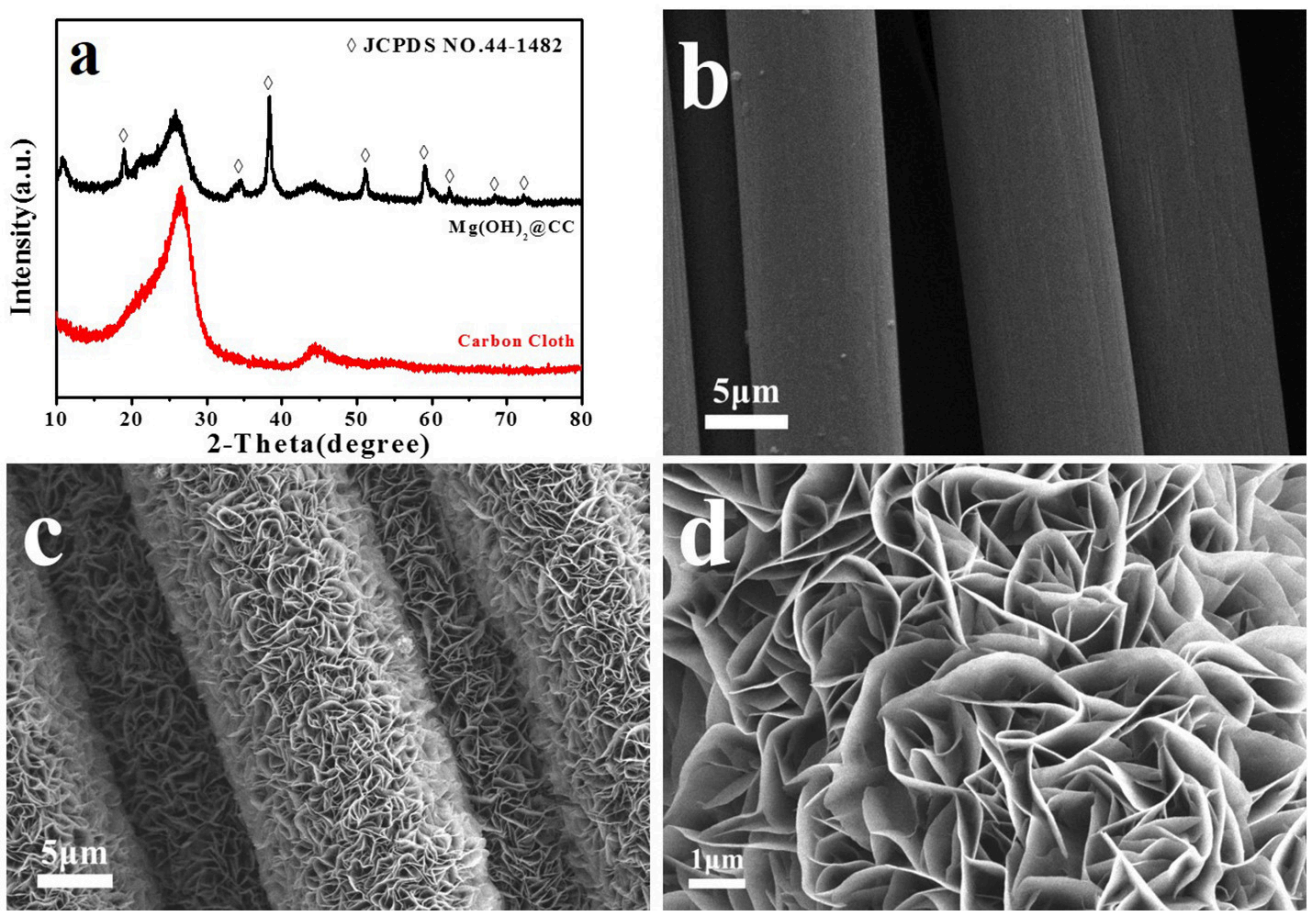
**(a)** XRD patterns of carbon cloth and Mg(OH)_2_@CC. **(b)** SEM image of the pure carbon cloth (^*^2000). **(c)** SEM image of Mg(OH)_2_@CC (^*^2000), **(d)** SEM image of Mg(OH)_2_@CC (^*^5000).

The XRD patterns of the carbon cloth and Mg(OH)_2_@CC composite are shown in Figure 1a, both of which exhibit two strong peaks at 2θ = 26.2 and 44.4°. The two peaks are attributed to (002) and (101) planes of the carbon cloth, respectively (Zhang G. et al., 2015). For the Mg(OH)_2_@CC composite, the characteristic diffraction peaks at 2θ = 18.4, 32.9, 38.0, 50.9, 58.7, 62.2, 68.4, and 72.1° (except peaks derived from the carbon cloth) are well indexed to (001), (100), (011), (102), (110), (111), (103), and (201) phases of Mg(OH)_2_, respectively (JCPDS NO.44-1482). Figure S2 shows the SEM image of Mg(OH)_2_@CC at different electrodeposition time. It can be found that with too short electrodeposition time, magnesium hydroxide on the surface of carbon cloth is very sparse. While with too long electrodeposition time, the magnesium hydroxide on the surface of carbon cloth will crack. Only with the appropriate electrodeposition time (around 10min), one layer of magnesium hydroxide is interlaced on the surface of the carbon cloth and attached strongly. Figures 1b–d show the typical morphologies of pure carbon cloth (^*^2000), Mg(OH)_2_@CC (^*^2000), and Mg(OH)_2_@CC (^*^5000), respectively. A low-magnification SEM image (Figure 1b) reveals that the fibers of the carbon cloth possess ~10 μm in diameter with a smooth surface. After the synthesis of Mg(OH)_2_@CC composites via electrodeposition, it is noticed that the interlaced nano-sheets of Mg(OH)_2_ are firmly and uniformly grown on the fibers with an average diameter of 15 μm (Figures 1c,d). The above results collectively demonstrated the successful integration of the Mg(OH)_2_@CC. In addition, the XRD patterns and SEM image of nano-Mg(OH)_2_ shown in Figure S3 indicated that the flower-like nano-Mg(OH)_2_ was successfully synthesized similar to the reported literature.

### Eu(III) adsorption isotherm and kinetics

**Figure 2:**
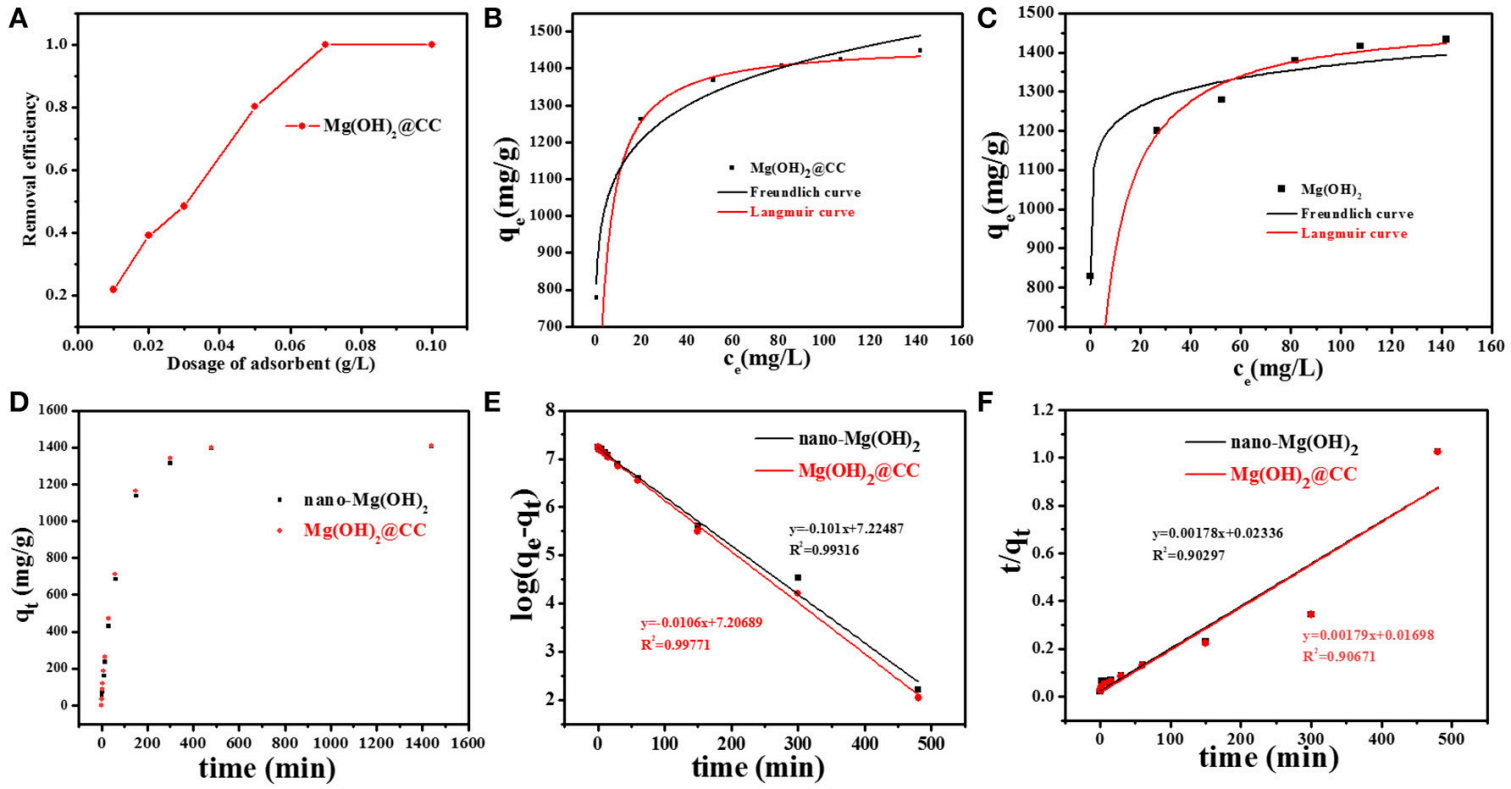
**(A)** Residual Eu(III) concentration in aqueous solution after adsorbed by Mg(OH)_2_@CC with different dosages. **(B,C)** Langmuir and Freundlich isotherms for Eu(III) adsorption by Mg(OH)_2_@CC and nano-Mg(OH)_2_. **(D)** Adsorption kinetics of Mg(OH)_2_@CC and nano-Mg(OH)_2_ toward Eu(III). **(E)** Pseudo-first-order kinetic model fitting for Eu(III) adsorption. **(F)** Pseudo-second-order kinetic model fitting for Eu(III) adsorption.

To confirm that the carbon cloth carrier in Mg(OH)_2_@CC has no negative effect on the adsorption performance of nano-Mg(OH)_2_, the adsorption effect of Mg(OH)_2_@CC toward Eu(III) was compared with nano-Mg(OH)_2_. Figure 2A shows that with an initial Eu(III) concentration of 100 mg/L (the concentration of REEs in the actual environment), Mg(OH)_2_@CC could decrease the residual Eu(III) concentration in the solution with increasing adsorbent dosage. When the dosage of adsorbent increased to 0.07 g/L, the removal efficiency can reach 99%. Once exceed 0.07 g/L, the adsorbent will be excessive. Therefore, in the experiments of adsorption kinetics and adsorption thermodynamics, the dosage of adsorbent was 0.07 g/L.

The adsorption isotherm data of nano-Mg(OH)_2_ and Mg(OH)_2_@CC to Eu(III) were fitted by the Langmuir and Feriundlich models. The results and related parameters are shown in Figures 2B,C and Table 1. Obviously, no matter using nano-Mg(OH)_2_ or Mg(OH)_2_@CC, the adsorption capacities increase with the concentration of Eu(III), until it reaches equilibrium. Both of Langmuir and Feriundlich models can be used to describe the adsorption of the two adsorbents (*R*^2^ > 0.95), while the *R*^2^ value of Langmuir model is higher than of Feriundlich model. Therefore, Langmuir model is more suitable to describe the adsorption of two adsorbents to Eu(III) and the adsorption type of two materials to Eu(III) is chemical adsorption. The maximum adsorption capacities of nano-Mg(OH)_2_ and Mg(OH)_2_@CC calculated by Langmuir model were 1428.571 and 1436.781 mg/g, respectively (Table 1). Therefore, the loading Mg(OH)_2_ on the carbon cloth will not affect the adsorption effect of Mg(OH)_2_.

**Table 1:** Parameters of Langmuir and Freundlich isotherm for Eu(III) on Mg(OH)_2_ and Mg(OH)2@CC.

**Eu(III)**	**Langmiur constant**			**Freundlich constant**		
	***R*^2^**	***q*_m_ (mg/g)**	***k*_L_**	***R*^2^**	***K*_F_(mg/g)**	***n***
Mg(OH)_2_	0.9995	1428.571	0.4375	0.9822	854.144	8.8261
Mg(OH)_2_@CC	0.9976	1436.781	0.2692	0.9631	1091.709	20.6612

The adsorption kinetics data of nano-Mg(OH)_2_ and Mg(OH)_2_@CC composites to Eu(III) were fitted by the pseudo-first-order kinetic and the pseudo-second-order kinetic model. The results and related parameters are shown in Figures 2D,E and Table 2. Figure 2D. showed that the equilibrium adsorption capacity of the two materials varies with time. When the dosage of adsorbent was 0.07 g/L and the initial concentration Eu(III) was 100 mg/L, both of the two adsorbents could reach equilibrium in 400 min and the equilibrium adsorption capacities were around 1,400 mg/g. Therefore, loading Mg(OH)_2_ on the carbon cloth will not affect its adsorption rate. The kinetic data of two adsorbents were fitted using the pseudo-first-order and pseudo-second-order models. The fitting results (Table 2) show that the kinetics of the two adsorbents were better described with pseudo-first-order model kinetics, which suggested that physical and chemical interactions may simultaneously contribute and control the uptake of Eu(III) onto the surface of Mg(OH)_2_.

**Table 2:** Kinetic parameters calculated from Pseudo-first order and Pseudo-second order kinetic models.

**Eu(III)**	**Pseudo-first order**			**Pseudo-second order**		
	***R*^2^**	***k*_1_(1/min)**	***q*_e_(mg/g)**	***R*^2^**	***k*_2_(1/min)**	***q*_e_(mg/g)**
Mg(OH)_2_	0.9938	0.0101	1373.201	0.9127	0.000138	555.56
Mg(OH)_2_@CC	0.9979	0.0106	1348.705	0.9164	0.000191	555.56

As a consequence, loading Mg(OH)_2_ on the surface of carbon cloth has no negative effect on its adsorption performance. The main reason is that the electrodeposited Mg(OH)_2_ is evenly distributed on the surface each carbon fiber. Since the carbon fibers are relatively independent, they have no coverage and interference to the active site of Mg(OH)_2_, leading to enough active sites exposing for Eu(III) adsorption.

### Material characterization after adsorption

**Figure 3:**
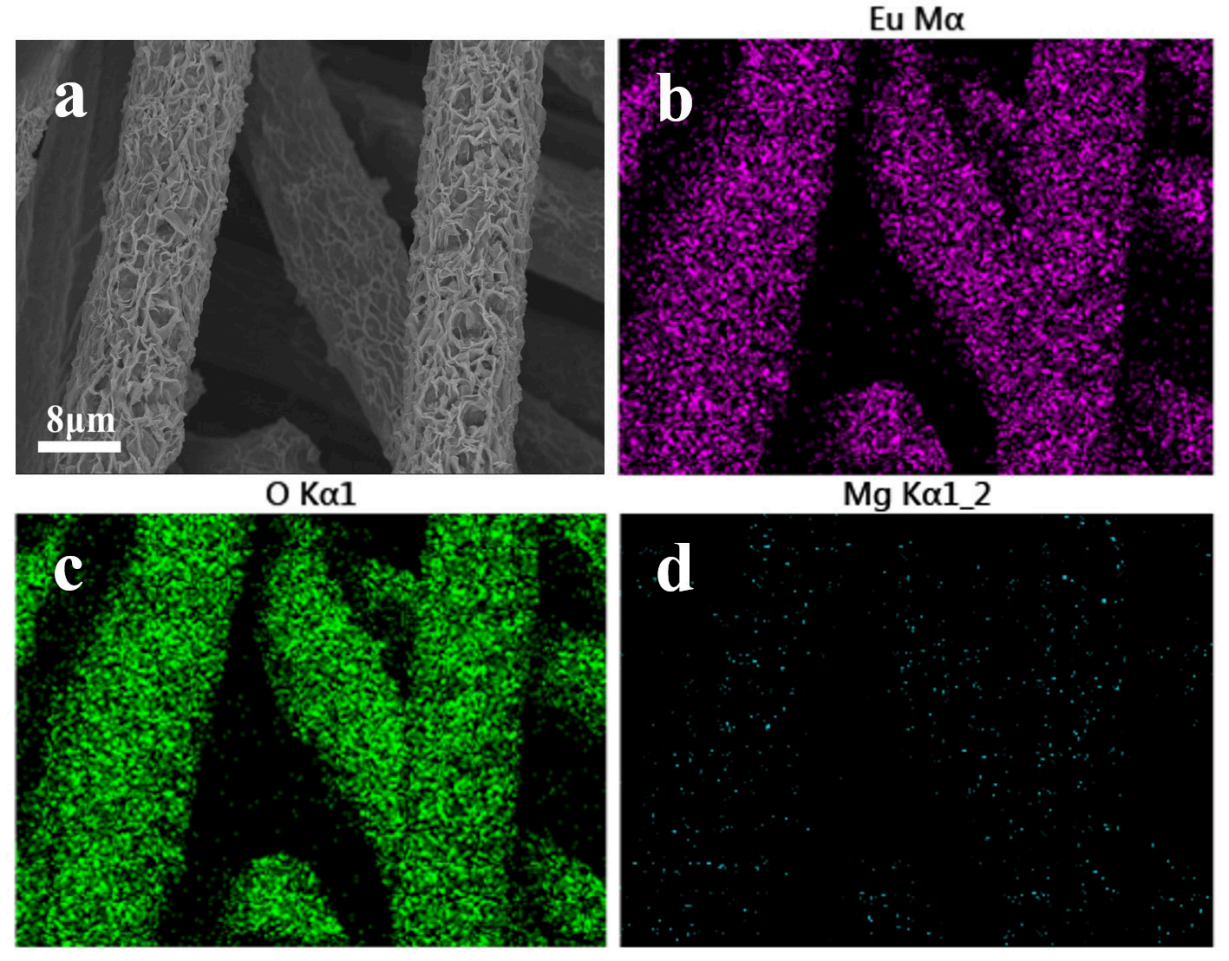
**(a)** SEM image of Mg(OH)_2_@CC-Eu after reacting with high concentration Eu(III) solution for 24 h. **(b)** Corresponding EDS elemental mapping images for Eu, **(c)** for O, **(d)** for Mg.

**Figure 4:**
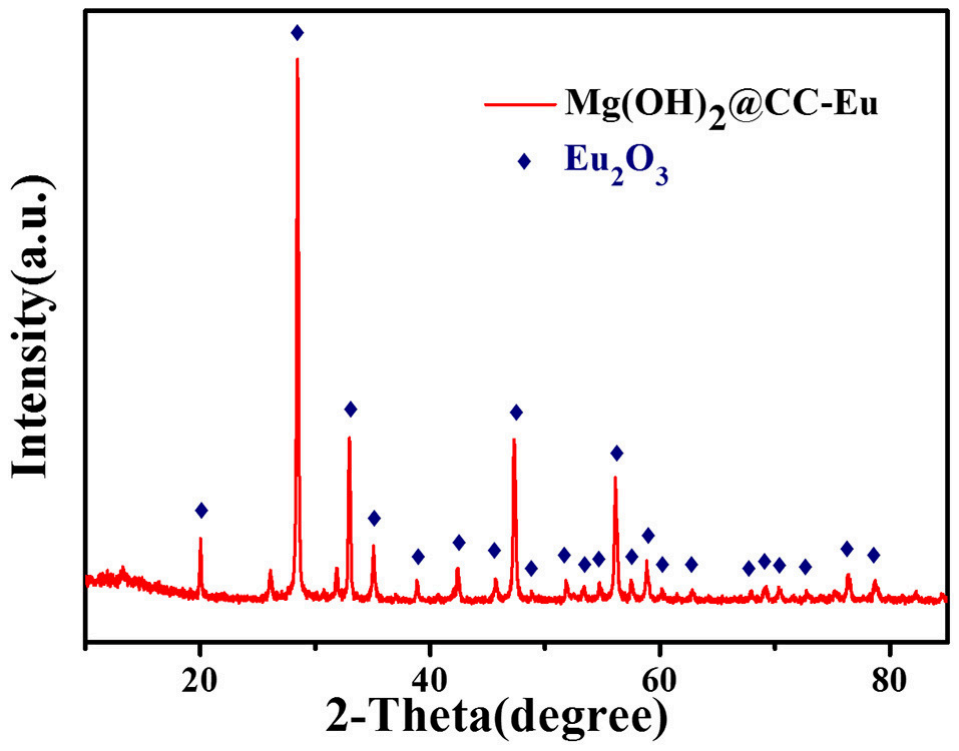
XRD patterns of Mg(OH)_2_@CC-Eu after calcination process without oxygen.

To explore the adsorption mechanism, Mg(OH)_2_@CC was exposed to Eu(III) solution with a high concentration. The morphology, distribution, and crystal phase of Mg(OH)_2_@CC-Eu(III) were determined by SEM-EDS and XRD. The SEM image showed that after adsorption, the original laminar structure of Mg(OH)_2_@CC slightly bended, but still retained the interlacing laminar structure (Figure 3a). This is due to the ions interaction between Eu(III) and Mg(II) that changes the morphology (Li et al., 2013). The distribution map of corresponding elements (Eu, O, Mg) was recorded by SEM-EDS (Figures 3b–d). The thick purple areas in Figure 3b show that the Eu is evenly distributed on the carbon fiber surface, while the sparse blue areas shown in Figure 3d indicate the decrease of Mg(II) content after adsorption. To confirm the enrichment of Mg(OH)_2_@CC to Eu(III), the adsorbed powder of Mg(OH)_2_-Eu was collected from the surface of carbon cloth through mechanical methods and calcinated at 800°C without oxygen. The XRD patterns of the obtained powder shown in Figure 4 can be well indexed as Europium Oxide phase (ICCD card no.00-034-0392). The corresponding ICP data are shown in Table 3. The content of europium in solid phase is 860,727 mg/kg, while the content of magnesium is only 21.3 mg/kg. According to ICP, SEM-EDS, and XRD data, it can be calculated that 99.66% of the obtained powder after calcination is europium oxide, while there is almost no magnesium oxide. Therefore, the Mg(OH)_2_@CC can extract Eu(III) from solution to the surface of the nanocomposite, which can realize the enrichment and recovery of REEs.

**Table 3:** ICP data of the reacted powder.

**Component**	**Mg**	**Eu**	**Eu_2_O_3_**
Conversion content	21.3 mg/kg	860, 727 mg/kg	996, 631 mg/kg

### Continuous system (fixed bed-column)

**Figure 5:**
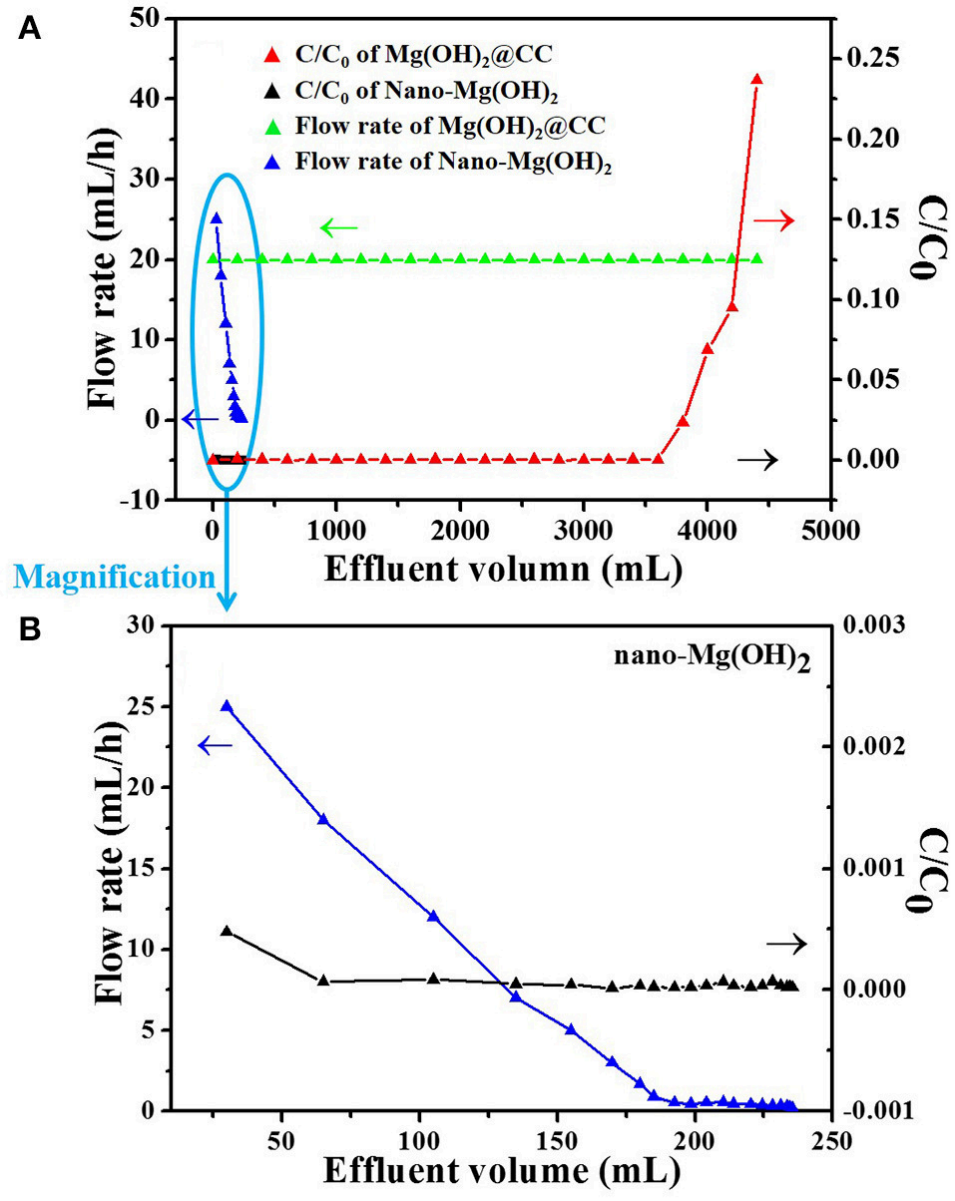
**(A)** Comparison curves of Eu(III) uptake in fixed-bed columns between nano-Mg(OH)_2_ and Mg(OH)_2_@CC (initial concentration of 100 mg/L Eu(III) effluent) **(B)** The magnification of the bottom left corner of **(A)**.

To further investigate the practical application of Mg(OH)_2_@CC, a fixed bed-column experiment was conducted to verify the removal performance of Eu(III) by Mg(OH)_2_@CC, taking nano-Mg(OH)_2_ as comparison. As shown in Figure 5, with the same quality of Mg(OH)_2_ and the initial Eu(III) concentration of 100 mg/L, the adsorption column performance of Mg(OH)_2_@CC is much better than that of pure nano-Mg(OH)_2_. It can be explained that the main adsorption mechanism of Mg(OH)_2_@CC to Eu(III) includes two parts: (1) ion exchange of Eu(III) with magnesium hydroxide; (2) the enhanced dispersion of nano-Mg(OH)_2_ by the surface function groups of carbon cloth. Under the two mechanisms, the treatment capacity of Mg(OH)_2_@CC is as high as 4,200 mL (C/C_0_ is < 0.10), while the concentration of effluent Eu(III) decreases to the μg/L level after treatment. Meanwhile, the effluent of Mg(OH)_2_@CC can be processed at a steady flow rate. The curve of nano-Mg(OH)_2_ toward Eu(III) uptaken in fixed-bed columns is shown in the bottom left corner of Figure 5A, where there is a short black curve. The magnification of the short black curve is shown in Figure 5B. The effluent effect of nano-Mg(OH)_2_ is superior (C/C_0_ approaches 0.00). However, with the increase of effluent volume, the flow rate also decreases rapidly. When the effluent volume reaches 240 mL, the flow rate is close to zero. A possible explanation is that the agglomeration of the nanoparticles reduces the specific surface area of nano-Mg(OH)_2_, and thus decreases the active sites for Eu(III) adsorption. Moreover, the agglomeration of nano-Mg(OH)_2_ leads to the increase of particle size, which further causes blockage in fix-bed column. This problem will severely limit the development of nano-Mg(OH)_2_ in practical application. Therefore, magnesium hydroxide plays an important role in the absorption of Eu(III) by Mg(OH)_2_@CC, while the main effect of carbon cloth is to strengthen the dispersion of nano-Mg(OH)_2_. The combination of nano-Mg(OH)_2_ with carbon cloth would further improve the efficiency of nano-Mg(OH)_2_ and prevent its loss.

## Conclusions

In summary, a new nanocomposite Mg(OH)_2_@ CC was prepared by electrodeposition, and used to adsorb Eu(III) in fixed-bed. The morphology and phase analyses revealed that the interlaced Mg(OH)_2_ nano-sheets are uniformly loaded on the carbon cloth fibers. The maximum adsorption capacity (1436.8 mg/g) and removal rate (95% within 300 min) of Mg(OH)_2_/CC toward Eu(III) is close to those of nano-Mg(OH)_2_, indicating that loading Mg(OH)_2_ on the surface of carbon cloth has no negative effect on its adsorption performance. Moreover, up to 99.66% of Eu_2_O_3_ is found in the adsorbed powder, indicating Mg(OH)_2_@CC could be an ideal candidate for the enrichment and recovery of Eu(III) from aqueous solution. In addition, Mg(OH)_2_@CC exhibited great potential for Eu(III) removal in the fixed-bed compared with nano-Mg(OH)_2_. Therefore, the excellent performance of Mg(OH)_2_@CC suggests that loading nanoparticles on CC via electrodeposition is a promising method to improve the application of nano-adsorption materials.

## Author contributions

YL: Organized the research problem on carbon cloth supported nano-Mg(OH)_2_ for the enrichment and recovery of Rare Earth Element Eu(III) from aqueous solution, and participated in writing the manuscript; CT: Synthesized Mg(OH)_2_@CC and nano-Mg(OH)_2_; SX: Conducted the batch adsorption experiments and the data fitting; YX: Conducted the characterization of the adsorbents before and after adsorption; RC: conducted the fixed bed experiment of two adsorbents; WL and ZL: Analyzed the mechanism of the carbon cloth on enhancing the adsorption ability of Mg(OH)_2_@CC toward Eu(III) in fixed bed and participated in writing the manuscript.

### Conflict of interest statement

The authors declare that the research was conducted in the absence of any commercial or financial relationships that could be construed as a potential conflict of interest.
