# Supplementary material for: Carbon Cloth Supported Nano-Mg(OH)2 for the Enrichment and Recovery of Rare Earth Element Eu(III) From Aqueous Solution
Source: Front Chem. 2018 Apr 18;6:118. doi: 10.3389/fchem.2018.00118 (PMC5915470; doi:10.3389/fchem.2018.00118)
Supplement: Supplementary file 1 [file Presentation1.pdf]

Supporting Information

Carbon Cloth Supported Nano-$Mg(OH)_2$ for the Enrichment and Recovery of Rare Earth Element Eu(III) from Aqueous Solution

Yinong, Li, [a] Chen Tian, [a] Weizhen, Liu, [a,*] Si Xu, [a] Yunyun, Xu, [a] Rongxin, Cui, [a] Zhang, Lin [a,]

a School of Environment and Energy, The Key Laboratory of Pollution Control and Ecosystem Restoration in Industry Clusters (Ministry of Education), South China University of Technology, Guangzhou, Guangdong 510006, China

Corresponding author:
Dr. Weizhen Liu
weizhliu@scut.edu.cn

1. The continuous effluent system flow diagram

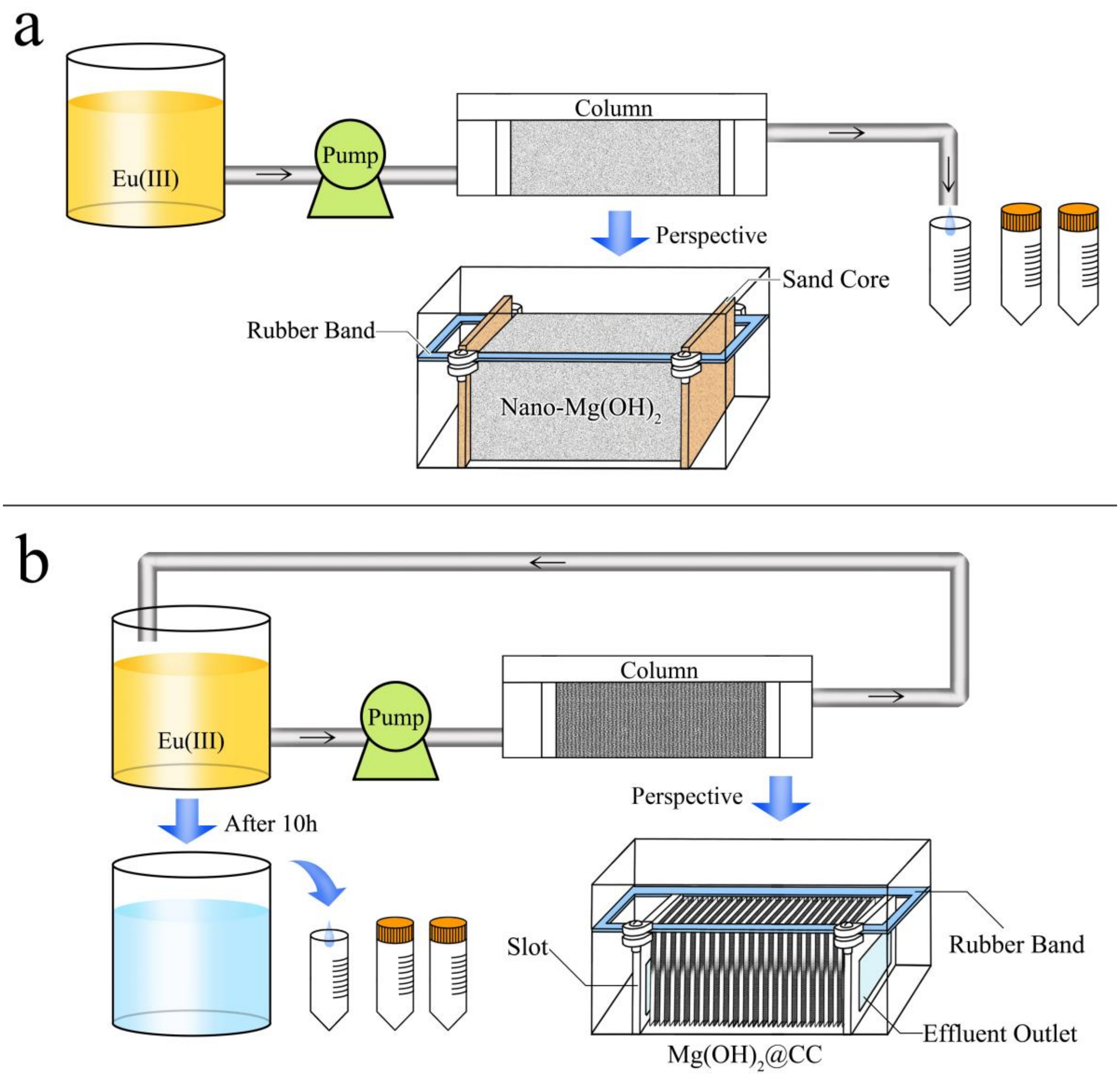

Fig. S1. The continuous effluent system flow diagram of (a) nano-$Mg(OH)_2$ (b) $Mg(OH)_2$@CC

2.SEM image of $Mg(OH)_2$@CC at different electrodeposition time

Figure S2 presents the SEM image of $Mg(OH)_2$@CC at different electrodeposition time, (a) 1min, (b) 10min, (c) 20min, (d)30min, respectively. In the shorter electrodeposition time, magnesium hydroxide on the surface of carbon cloth is very sparse, while in the longer electrodeposition time, the magnesium hydroxide on the surface of carbon cloth will crack. In the appropriate electrodeposition time, only one layer of magnesium hydroxide, which is interlaced on the surface of the carbon cloth, is very strong.

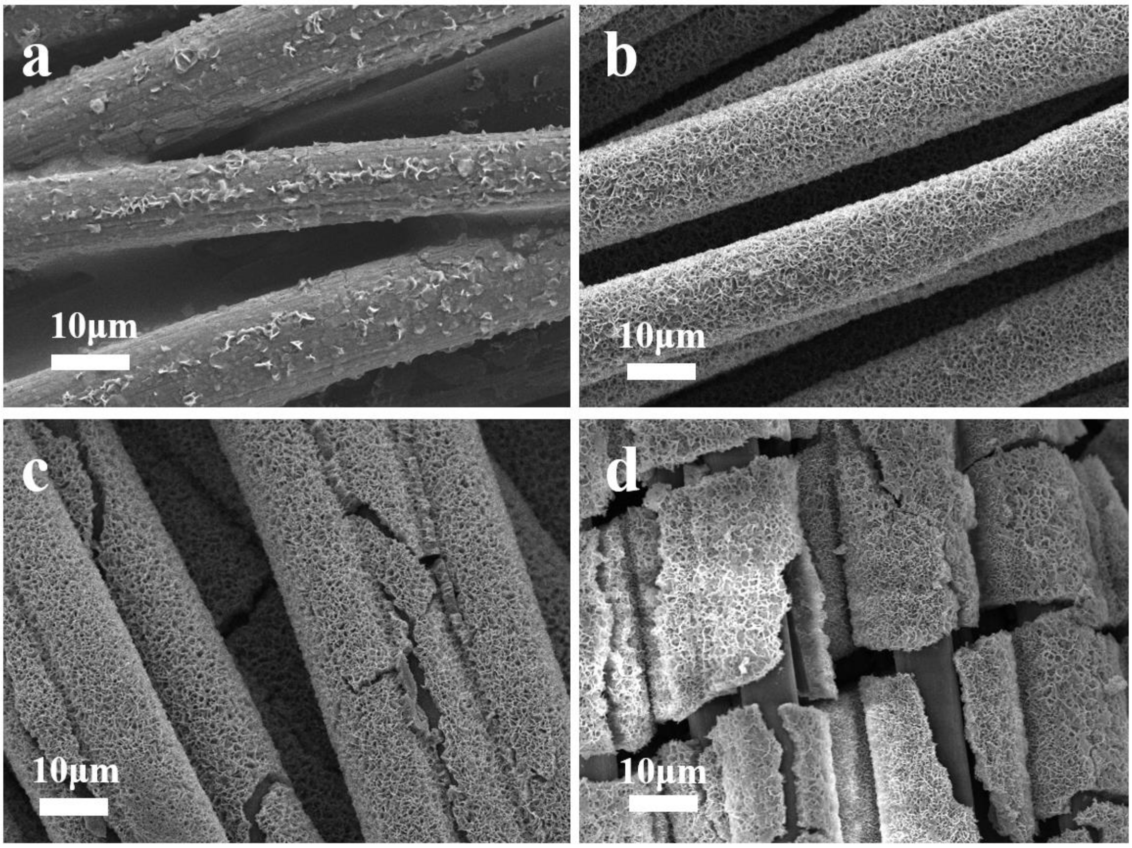

Fig. S2. SEM image of $Mg(OH)_2$@CC at different electrodeposition time, (a) 1min, (b) 10min, (c) 20min, (d)30min

3.the XRD patterns and SEM image of nano-$Mg(OH)_2$

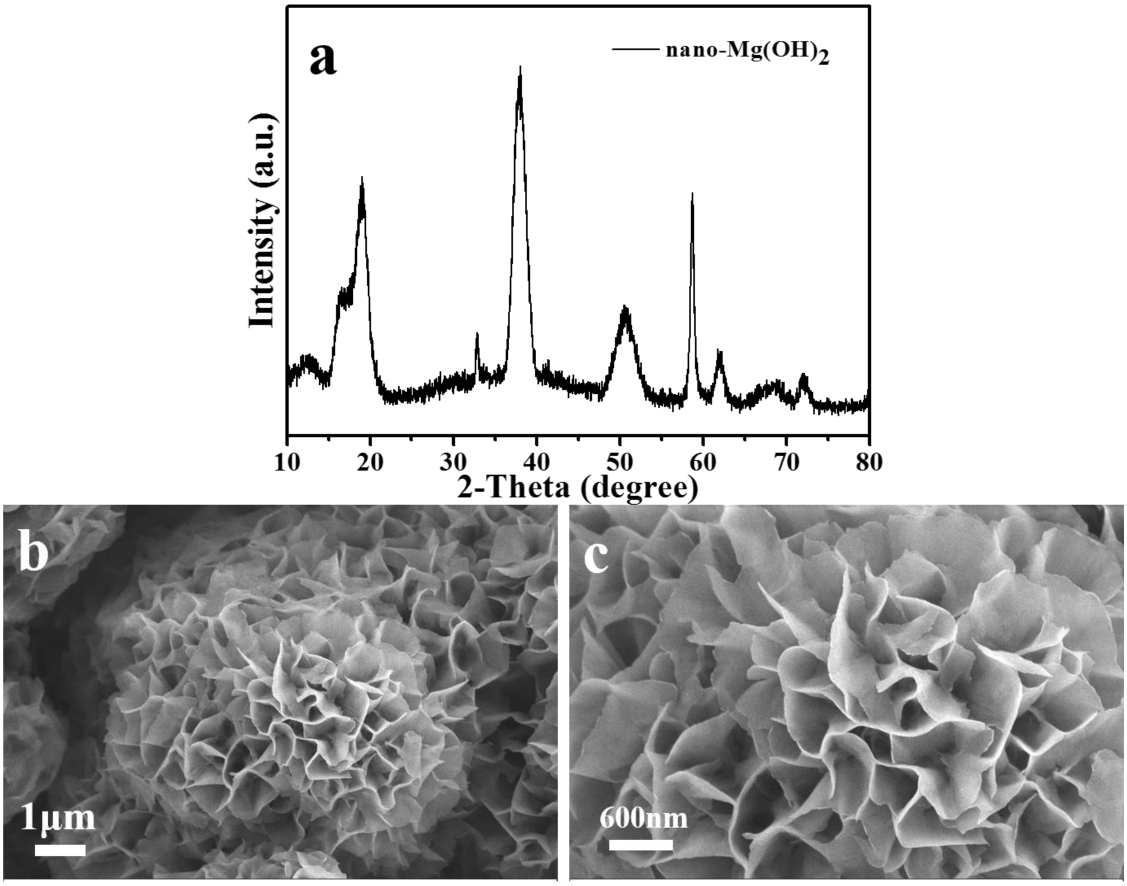

Fig. S3. (a) XRD patterns of nano-$Mg(OH)_2$, (b) SEM image of nano-$Mg(OH)_2$ (*10000), (c) SEM image of nano-$Mg(OH)_2$ (*20000)
